# Biosynthesis of sulfonamide and sulfamate antibiotics in actinomycete

**DOI:** 10.1093/jimb/kuab001

**Published:** 2021-01-29

**Authors:** Takayoshi Awakawa, Lena Barra, Ikuro Abe

**Affiliations:** Graduate School of Pharmaceutical Sciences, The University of Tokyo, Bunkyo-ku, Tokyo 113-0033, Japan; Collaborative Research Institute for Innovative Microbiology, The University of Tokyo, Yayoi 1-1-1, Bunkyo-ku, Tokyo 113-8657, Japan; Graduate School of Pharmaceutical Sciences, The University of Tokyo, Bunkyo-ku, Tokyo 113-0033, Japan; Graduate School of Pharmaceutical Sciences, The University of Tokyo, Bunkyo-ku, Tokyo 113-0033, Japan; Collaborative Research Institute for Innovative Microbiology, The University of Tokyo, Yayoi 1-1-1, Bunkyo-ku, Tokyo 113-8657, Japan

**Keywords:** Sulfonamide, Sulfamates, Actinomycete, Bioactivity, Biosynthesis

## Abstract

Sulfonamides and sulfamates are a group of organosulfur compounds that contain the signature sulfamoyl structural motif. These compounds were initially only known as synthetic antibacterial drugs but were later also discovered as natural products. Eight highly potent examples have been isolated from actinomycetes to date, illustrating the large biosynthetic repertoire of this bacterial genus. For the biosynthesis of these compounds, several distinct and unique biosynthetic machineries have been discovered, capable to generate the unique S–N bond. For the creation of novel, second generation natural products by biosynthetic engineering efforts, a detailed understanding of the underlying enzyme machinery toward potent structural motifs is crucial. In this review, we aim to summarize the current state of knowledge on sulfonamide and sulfamate biosynthesis. A detailed discussion for the secondary sulfamate ascamycin, the tertiary sulfonamide sulfadixiamycin A, and the secondary sulfonamide SB-203208 is provided and their bioactivities and mode of actions are discussed.

## Introduction

**Fig. 1.**
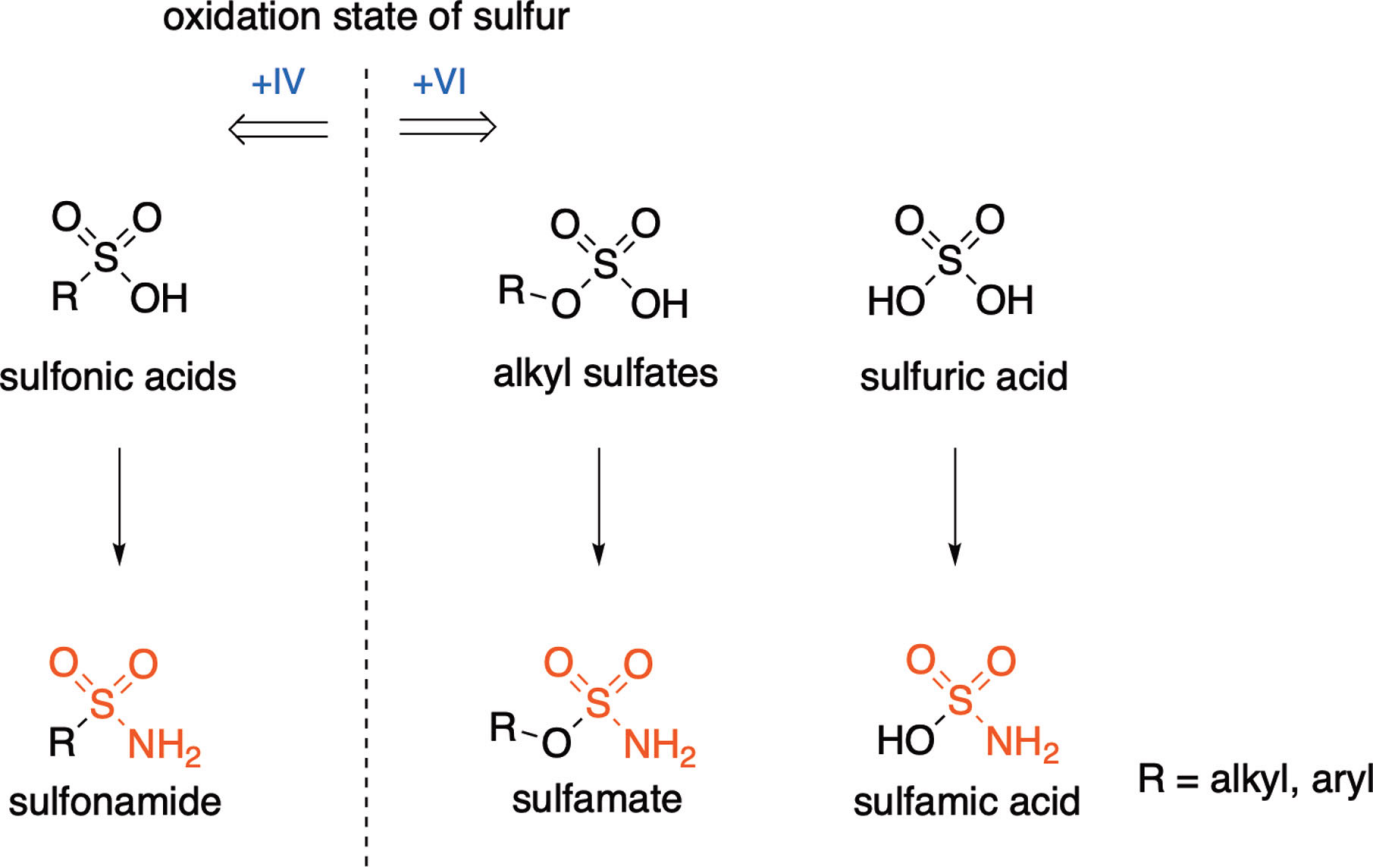
Structural relationships of sulfonamides and sulfamates.

Organosulfur compounds exhibit unique chemical properties due to the features of the inherent sulfur heteroatom and the arising reactivity. Sulfur is a third-period element and occupies the 3s, 3p, and sometimes 3d orbitals that are significantly larger than the second period elements orbitals (2s, 2p) such as oxygen or nitrogen. Additionally, sulfur is much less electronegative and organosulfur compounds with oxidation state +II, +IV, and +VI and coordination numbers from 0 to 7 are known (Block, 1978). Sulfonamides and sulfamates are organosulfur compounds that carry a sulfamoyl group represented by a tetrahedral sulfur atom connected to two oxygen, and one amine functionality (R^1^SO_2_NR^2^_2_, R^1^ = alkyl, aryl, R^2^ = alkyl, aryl, or H). Whereas the sulfur atom is linked to an alkyl residue via a S–C bond for sulfonamides, sulfamates exhibit an S–O bond connecting the sulfamoyl group to an alkoxy residue. Sulfonamides are the amido analogs of sulfonic acids and the sulfur atom exhibits a +IV oxidation state. Sulfamates, on the other hand, can be regarded as the alkyl esters of sulfamic acid and are therefore the amido analogs of alkyl sulfates, exhibiting a sulfur atom in oxidation state +VI (Fig. 1).

**Fig. 2.**
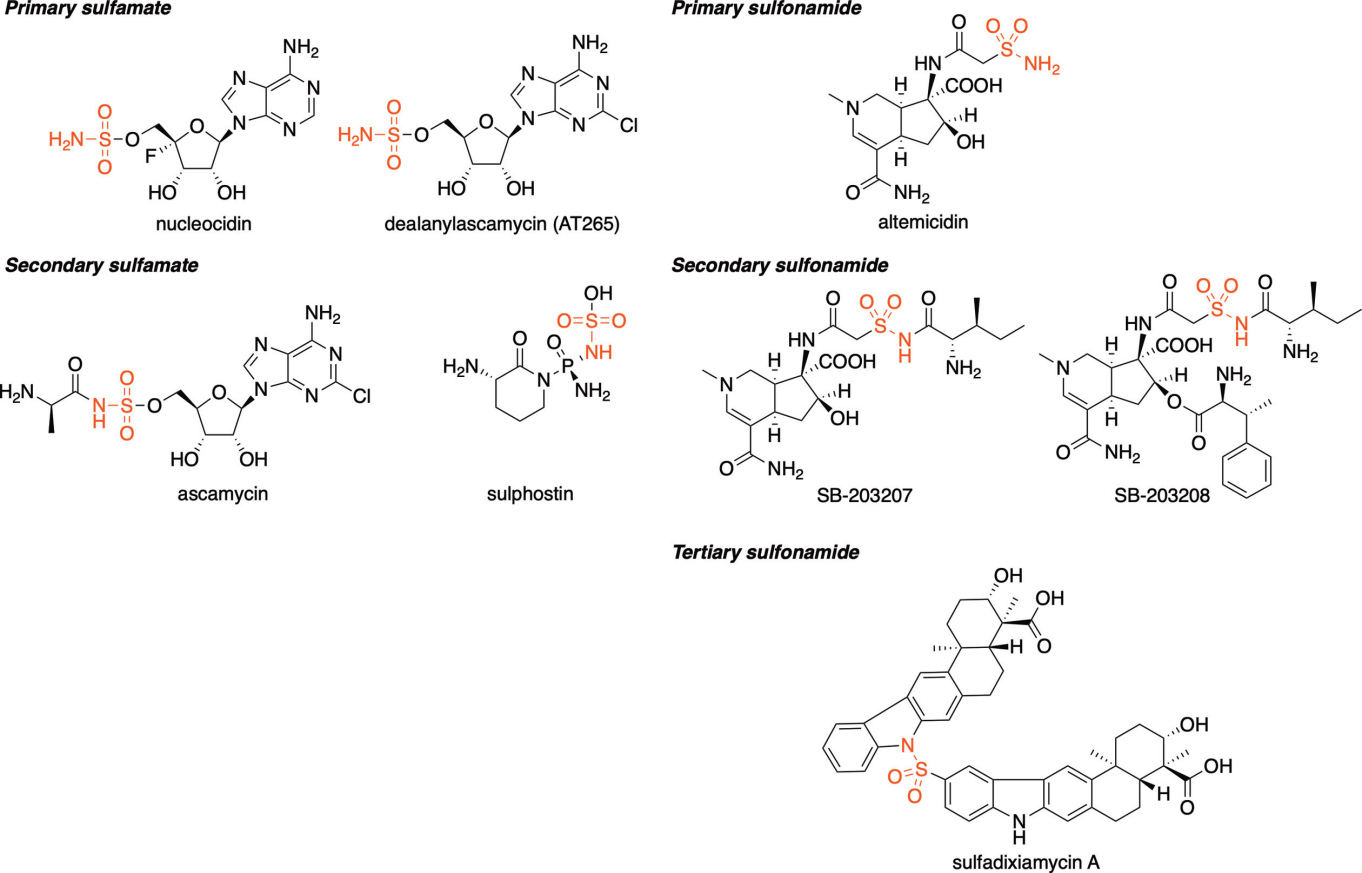
The structures of sulfamate and sulfonamide compounds from actinomycete.

Sulfonamides and sulfamates constitute a diverse family of highly pharmacologically active compounds and many clinically used drugs contain the signature sulfamoyl structural motif. Sulfonamides are widely known as synthetic “sulfa drugs”, and were the first chemotherapeutically used antibacterial compounds, represented by the most famous drug Prontosil (Brooks, 1989). Prontosil structurally resembles *p*-aminobenzoic acid, the native substrate of the dihydropteroate synthase, a key enzyme in bacterial folate biosynthesis (Ferone, 1977). A plethora of synthetic sulfamoyl containing compounds have been synthesized and drugs functioning as diuretics, uricosurics, hypoglycemic treatments, or antimicrobial have been developed (Brooks, 1989). While sulfonamides and sulfamates are mainly known as synthetic compounds, intriguingly, several examples were also discovered from natural sources (Petkowski et al., 2018). The majority of these secondary metabolites are produced by actinomycetes, and primary sulfamates such as nucleocidin and dealanylascamycin (AT265), the primary sulfonamide altemicidin, the secondary sulfamates, ascamycin and sulphostin, the secondary sulfonamides SB-203207 and SB-203208, and the tertiary sulfonamide sulfadixiamycin A have been isolated (Fig. 2). Since the revolution of natural product sciences, caused by the development of genome and transcriptome sequencing technologies, the investigation and discovery of the genetic and biochemical basis of natural products biosynthesis opened the door for the development of novel drugs and drug leads by biosynthetic engineering. Given the pharmaceutical importance of sulfamoyl containing compounds such as sulfonamides and sulfamates, we here aim to summarize the current state of knowledge on sulfonamide and sulfamate biosynthesis in actinomycete. As the main topic of this review is the biosynthetic reaction for S–N bond formation, see Waldman et al. (2017) for the overview of heteroatom–heteroatom bond forming reaction in nature.

## Biosynthesis of Sulfamate Compounds

**Fig. 3.**
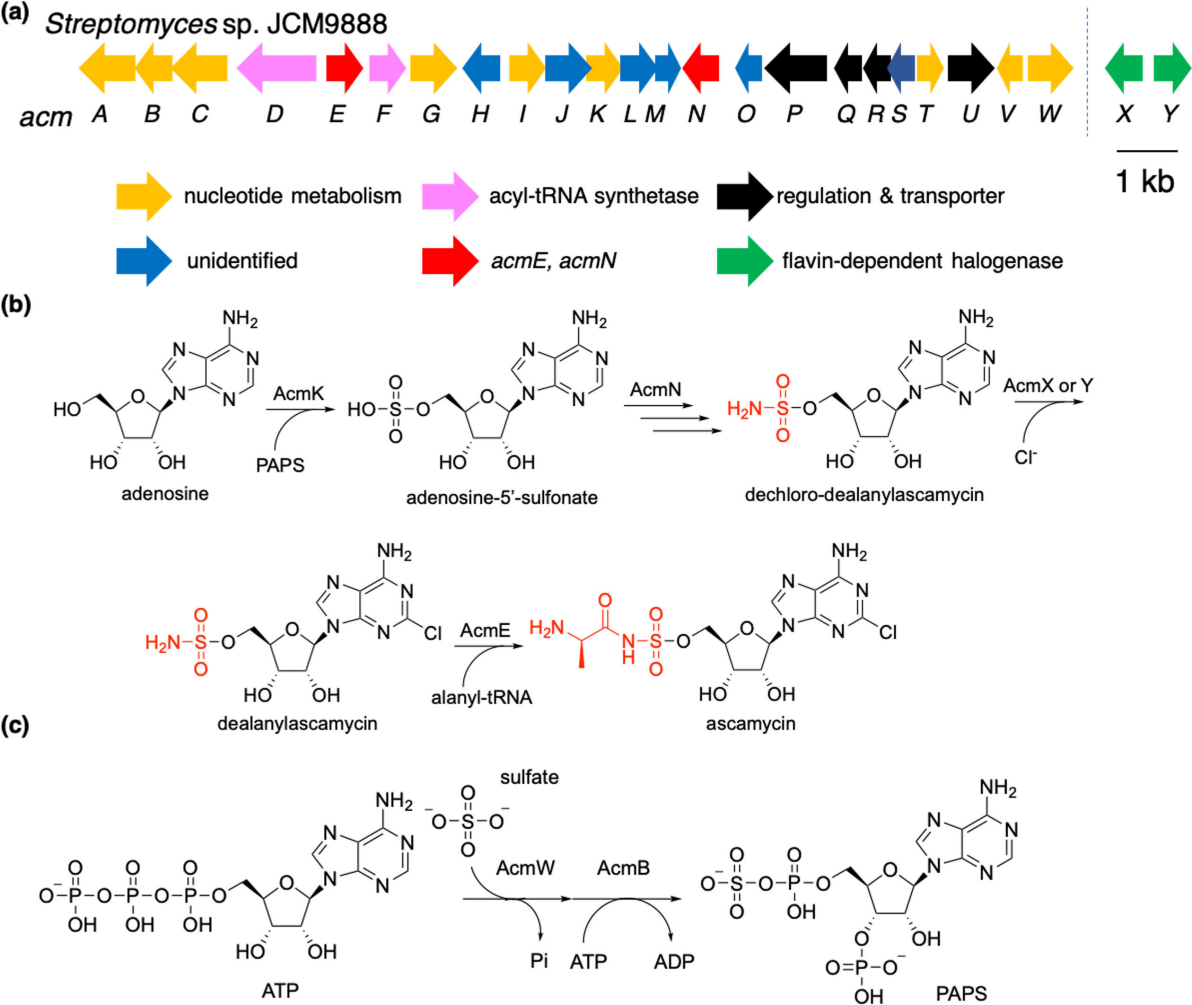
The proposed ascamycin biosynthesis. (a) The organization of *acm* cluster. (b) The biosynthesis of ascamycin from adenosine. (c) The biosynthesis of PAPS from ATP.

Dealanylascamycin, ascamycin, and nucleocidin are rare 5′-*O*-sulfamate nucleoside antibiotics isolated from *Streptomyces* sp. JCM9888 (Isono et al., 1984; Morton et al., 1969; Takahashi & Beppu, 1982) (Fig. 3). Whereas dealanylascamycin and nucleocidin possess a primary sulfamate moiety, ascamycin carries an additional *N*-l-alanyl residue and can therefore be described as a secondary sulfamate. Notably, ascamycin and dealanylascamycin exhibit differing antimicrobial activities. Ascamycin possesses limited antibiotic properties and is active toward only a few bacterial species like *Xanthomonas*. Dealanylascamycin, on the other hand, exhibits a broader antibacterial spectrum against both gram-positive and gram-negative bacteria. Additionally, dealanylascamycin was found to have anti-trypanosomal and -amoebae activities. The detailed investigation of their mode of action showed that both compounds dealanylascamycin and ascamycin are able to inhibit protein synthesis by using a cell-free protein translation assay system (Osada & Isono, 1985). The other study revealed that ascamycin is converted to dealanylascamycin by a hydrolyzing aminopeptidase in *Xanthomonas campestris* pv*. citri* (Osada & Isono, 1986), and the authors proposed that the differing antimicrobial properties arise from differences in the ability to penetrate the cell membrane of target organisms, as only dealanylascamysin can be transferred into the cell. However, the target of dealanylascamycin in protein synthesis has not been identified.

**Fig. 4.**
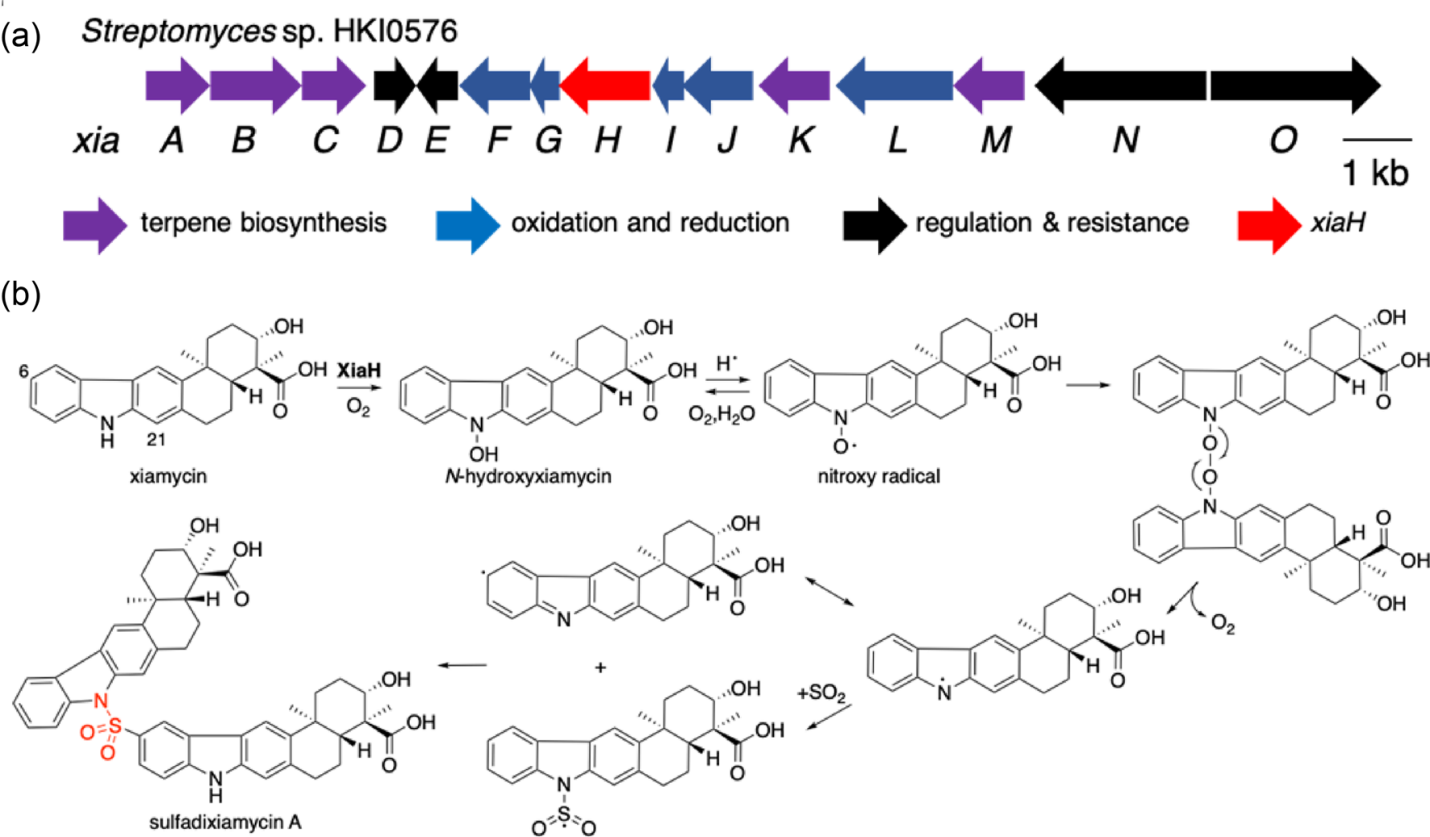
The biosynthesis of sulfadixiamycin A. (a) The organization of *xia* gene cluster. (b) *N*-hydroxylation-triggered dimerization of xiamycin via SO_2_ by the flavin-binding enzyme XiaH.

The cosmid harboring the biosynthetic gene cluster (*acm* cluster, Fig. 3a) for ascamycin biosynthesis was searched by using *acmG* (sulfatase) as a query, and the gene cluster was confirmed by gene inactivation of *acmG* and *acmK* (sulfotransferase) (Zhao et al., 2014). In initial studies, the research group analyzed the function of AcmE, predicted as an esterase with weak homology to the identified aminopeptidase which hydrolyzes ascamycin to produce dealanylascamycin in *Xanthomonas citri* (Osada & Isono, 1985). The *acmE*-inactivated mutant no longer produced ascamycin, but dealanylascamycin, suggesting the function of AcmE in the amidation of dealanylascamycin with the l-alanyl moiety in the late stage of the biosynthetic pathway (Fig. 3b). The l-alanyl moiety was hypothesized to be derived from alanyl-tRNA, since *acmE* is flanked by two acyl-tRNA synthetase genes (*acmD* and *F*); however, no biochemical evidence has been reported. The source of the sulfamoyl group has been proposed to be derived from a sulfate group, followed by its conversion into a sulfamate functionality. The first step was suggested to be catalyzed by the 3′-phosphoadenosine 5′-phosphosulfate (PAPS)-dependent sulfotransferase AcmK. PAPS-dependent sulfotransferases are a large family of enzymes that transfer a sulfuryl group from the donor co-substrate PAPS to a nucleophilic acceptor molecule. This common strategy for sulfo-group transfer is shared in many biosynthetic pathways and sulfotransferases acting on steroids, bioamines, therapeutic drugs, glucosaminylglycans, as well as proteins are known (Negishi et al., 2001).

In the ascamycin gene cluster, PAPS itself in thereby suggested to be generated by the ATP sulfurylase AcmW and the adenylylsulfate kinase AcmB from two molecules of ATP and sulfate (Fig. 3c) (Negishi et al., 2001; Petkowski et al., 2018). The proposed adenosine-5′-sulfonate intermediate is then further converted to dechloro-dealanylascamycin. Based on gene annotation and bioinformatic predictions, the authors hypothesized that the amidinotransferase AcmN is responsible for the S–N bond formation event to yield the primary sulfamate. However, the sulfamate formation is highly likely to involve at least a second enzyme to catalyze the necessary modification of the attached amidino group, considering the catalysis of amidinotransferase (Humm et al., 1997). The following halogenation was suggested to be catalyzed by the flavin-dependent halogenases AcmX or Y. Apart from the initial report on the identification of the *acm*-gene cluster (Zhao et al., 2014), no detailed biochemical investigation on the function of the involved enzymes has been reported.

Nucleocidin contains a rare fluorine atom at the C-4′ position in the nucleoside core of ascamycin (Feng et al., 2019; Morton et al., 1969). The target of nucleocidin is also the bacterial protein biosynthesis similarly to dealanylascamycin, and it was shown to inhibit the incorporation of aminoacyl-tRNA into the ribosome (Florini et al., 1966). Interestingly, nucleocidin was produced in *Streptomyces calvus* ATCC13382, only when a rare codon, Leu-tRNA^UUA^ (*bldA*) was supplied. Its gene cluster (*nuc* cluster) was identified through gene deletion (Zhu et al., 2015). Apart from early feeding experiments that showed incorporation of labeled glycerol into position C-5′ of nucleocidin, (Feng et al., 2017), there is little knowledge on its biosynthesis. Considering that NucN, an ORF that has 48.7% similarity to AcmN, is encoded in *nuc* cluster, nucleocidin likely shares the same S–N bond-forming mechanism with ascamycin biosynthesis. Apart from the cryptic sulfamate installation, the introduction of fluorine and how the C–F bond is formed also remains enigmatic. A known mechanism for fluoride incorporation into SAM by 5′-fluoro-5′-deoxyadenosine synthase flA from the actinomycete *Streptomtyces cattleya* (O'Hagan et al., 2002) has been reported, but no *flA* homolog is encoded in the *nuc* cluster, suggesting a novel mechanism for C–F bond formation in nucleocidin biosynthesis.

Another sulfamate compound, sulphostin was isolated from *Streptomyces* sp. MK251-43F3 by the Institute of Microbial Chemistry (Akiyama et al., 2001). This compound exhibits strong inhibitory activity against dipeptidyl peptidase IV that is a cell-surface serine peptidase that cleaves a dipeptide from the N-terminus of target proteins (De Meester et al., 1999). Because dipeptidyl peptidase IV controls the insulin release and modulation of thymocyte activity, sulphostin likely possesses therapeutic potential for type II diabetes and immune disorders. From its structure, the biosynthetic precursor can be expected to be l-ornithine, however nothing is known about the biosynthesis of sulphostin.

## Biosynthesis of Sulfadixiamycin A

Xiamycins are rare indole alkaloids with anti-HIV activities that possess a unique cyclic sesquiterpenoid structure and were isolated from mangrove-derived *Streptomyces* sp. HKI0576 (Ding et al., 2010) and *Streptomyces pactum* SCSIO 02999 (Zhang et al., 2012). The two biosynthetic clusters for xiamycin were identified in these two strains by Hertweck's group (Xu et al., 2012) and by Zhang's group (Li et al., 2012), respectively (*xia* cluster, the same name for these two clusters) (Fig. 3a). Heterologous expression of the *xia* gene cluster in *Streptomyces albus* by Hertweck's group led to production of several minor products including xiamycin dimers, named dixamycins, connected via a N–C or N–N bond (Baunach et al., 2013). The promising antibacterial activity of dixiamycin prompted the researchers to further investigate the minor constituents from a 50 L culture of the *S. albus* expression strain. Three novel xiamycin analogs, possessing a unique sulfonyl-linkage between N-1′ and C-6 were isolated and named sulfadixiamycin A–C (Baunach et al., 2015). These compounds exhibit potent antibacterial activity against *Bacillus subtilis, Staphylococcus aureus*, and MRSA. The biological target of the sulfadixiamycines is still unknown, but given the unique structural features of these compounds, a distinct mechanism to other sulfa drugs seems likely.

In order to identify the key biosynthetic enzyme responsible for the S–N bond formation, the authors deleted several ORFs encoding oxygenases in the *xia* cluster and deletion of *xiaH* led to a completely abolished production of dixiamycins including sulfadixiamycin A–C, while it still produced xiamycin. This data clearly indicated that XiaH is responsible for the dimerization of xiamycin. XiaH is a flavin-binding oxygenase and the authors proposed a radical-initiated reaction mechanism and involvement of sulfite-derived sulfur dioxide (Baunach et al., 2015). Zhang's group characterized the direct product of XiaH (XiaK in their gene cluster) as *N*-hydroxyxiamycin, and it spontaneously turned to be *N*-hydroxy radical adduct, in the mechanistic study *in vitro* (Zhang et al., 2017). Based on the results from these two experiments, the reaction mechanism for sulfadixiamycin A biosynthesis can be proposed as below.

In the initial step, XiaH catalyzes *N*-hydroxylation, and the resultant *N*-hydroxyxiamycin spontaneously generates a nitroxy radical (Fig. 4). The nitroxy radical undergoes dimerization accompanied with deoxygenation to yield a nitrogen radical. The nitrogen radical reacts with sulfur dioxide and yields a sulfonyl radical that upon recombination with another xiamycin radical builds up the N–S–C bond characteristic for sulfadixiamycin A. This radical reaction is reminiscent of synthetic methods for the polymerization of styrene and sulfur dioxide (Barb, 1952; Matsuda et al., 1972), and an intriguing method by Nature to increase the complexity of natural products. In sulfadixiamycin B and C, the sulfur is bound to C-6 or C-21 of the aromatic indole moiety via C–S–C bond, indicating different resonance stabilized radical intermediates.

## Biosynthesis of Altemicidin, SB-203207, and SB-203208

**Fig. 5.**
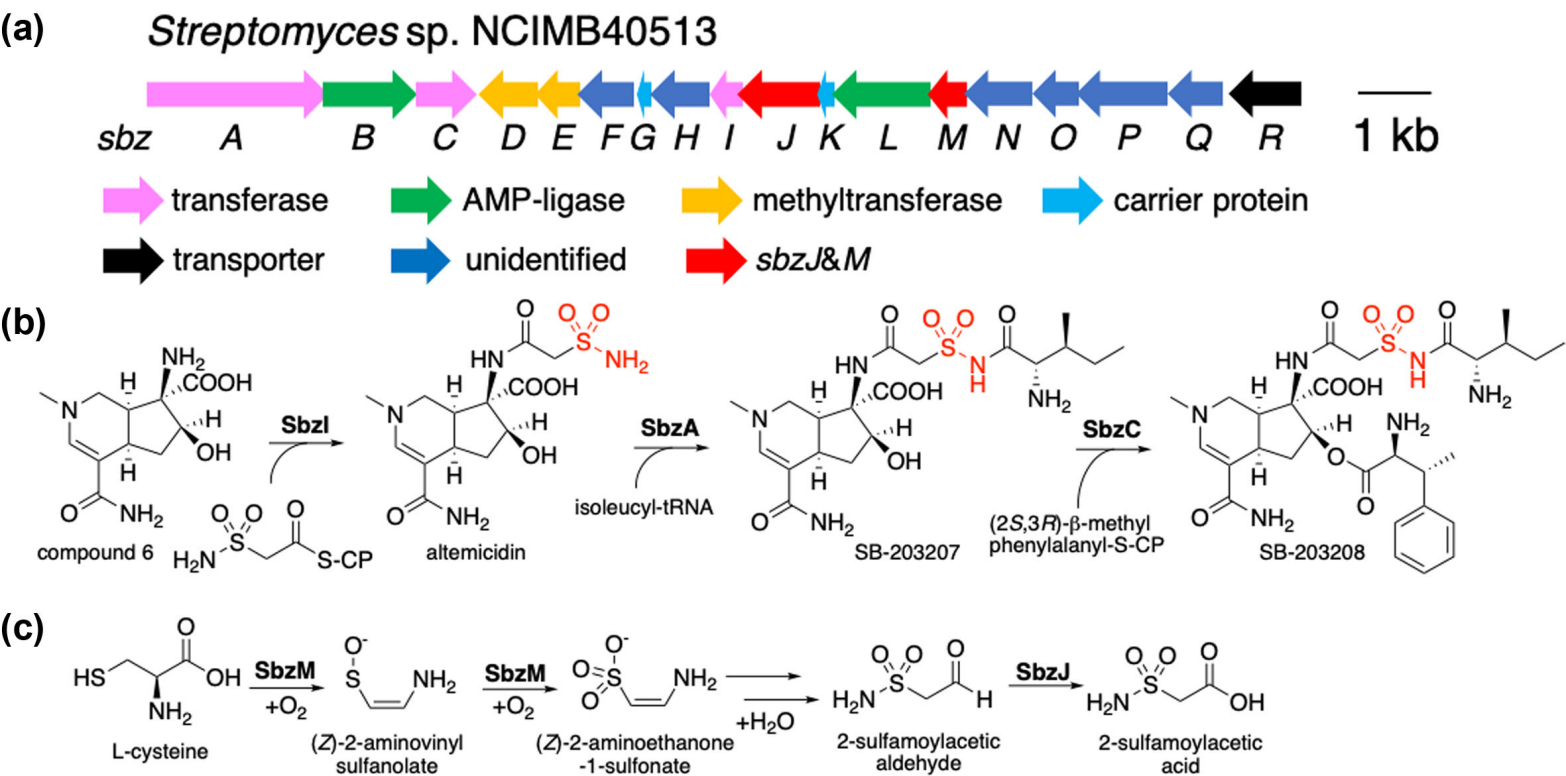
The biosynthesis of SB-203208. (a) The organization of *sbz* cluster. (b) The aminoacyl-transfer reactions to produce SB-203208. (c) The multistep oxygenation catalyzed by SbzM and SbzJ to produce 2-sulfamoylacetic acid from l-cysteine.

In 1989, altemicidin was isolated from the marine actinomycete *Streptomyces sioyaensis* SA-1758 in the course of a screening program for novel acaricidal substances (Fig. 5a) (Takahashi et al., 1989). Altemicidin exhibits antitumor activity, as well as antibacterial activity specific toward *Xanthomonas* species, similar to ascamycin. The structure of altemicidin was determined as (1*R*,2*S*,3a*R*,7a*S*)-4-carbamoyl-2-hydroxy-6-methyl-1-(sulfamoylacetamido)-2,3,3a,6,7,7a-hexahydro-6-azainden-1-carboxylic acid, by NMR and X-ray crystallographic analyses after derivatization with 9-hydroxyxanthene (Takahashi et al., 1989). In 2000, the two related compounds SB-203207 and SB-203208 were isolated from the soil actinomycete *Streptomyces* sp. NCIMB 40513 during a metabolite screening for new inhibitors of isoleucyl-tRNA synthetase (Houge-Frydrych et al., 2000; Stefanska et al., 2000). SB-203207 was found to inhibit the isoleucyl-tRNA synthetases from *Staphylococcus, Pseudomonas, Candida*, and Rat liver at IC_50_ < 2 nM, while showing only weak inhibition of the isoleucyl-tRNA synthetase from the producer strain (IC_50_ = 401 nM). This data implies the existence of a self-resistance mechanism in the SB-203207 producer strain. The proteins responsible for self-resistance against antibiotics are usually efflux pumps, modification enzymes, and homologs of target proteins with specific mutations to inhibit binding of the produced antibiotic. In recent years, the self-resistance gene-guided search for biosynthetic gene clusters has been demonstrated as a powerful tool (Tang et al., 2015; Yan et al., 2018, 2020).

The SB-203208 biosynthetic gene cluster (*sbz* cluster) that contained genes encoding a putative self-resistance-related isoleucyl-tRNA synthetase (*sbzA*), MppJ-like methyltransferase (Huang et al., 2009) (*sbzD*), two sets of AMP-ligases (*sbzBL*), and two carrier proteins (*sbzGK*), was found in the genome sequence of *Streptomyces* sp. NCIMB40513 (Hu et al., 2019). The heterologous expression of the *sbz* cluster in *Streptomyces lividans* yielded the target compounds altemicidin (10 mg/l), SB-203207 (1.1 mg/l), and SB-203208 (1.8 mg/l). The primary sulfonamide group in altemicidin was shown to be derived from l-cysteine by feeding experiments and the key biosynthetic enzymes were identified to be a cupin oxygenase family enzyme (SbzM) and an NAD^+^-dependent aldehyde dehydrogenase (SbzJ) by gene inactivation. *In vitro* assays with SbzM and l-cysteine revealed a highly unusual oxidative rearrangement toward 2-sulfamoylacetic aldehyde. To monitor the reaction, two different labeling agents for carboxyl and aldehyde groups were utilized, as well as direct ^13^C-NMR analysis of the SbzM reaction employing (^13^C_3_, ^15^N_1_)cysteine. In the subsequent reaction, SbzJ was shown to oxidize the resulting aldehyde to the corresponding 2-sulfamoylacetic acid (Fig. 5b). The 2-sulfamoylacetic acid moiety is incorporated into the side chain of altemicidin by the action of a Gcm5-related *N*-acetyltransferase-like enzyme (SbzI). The altemicidin biosynthetic pathway was the first example of a cupin oxygenase-type derived sulfonamide.

**Fig. 6.**
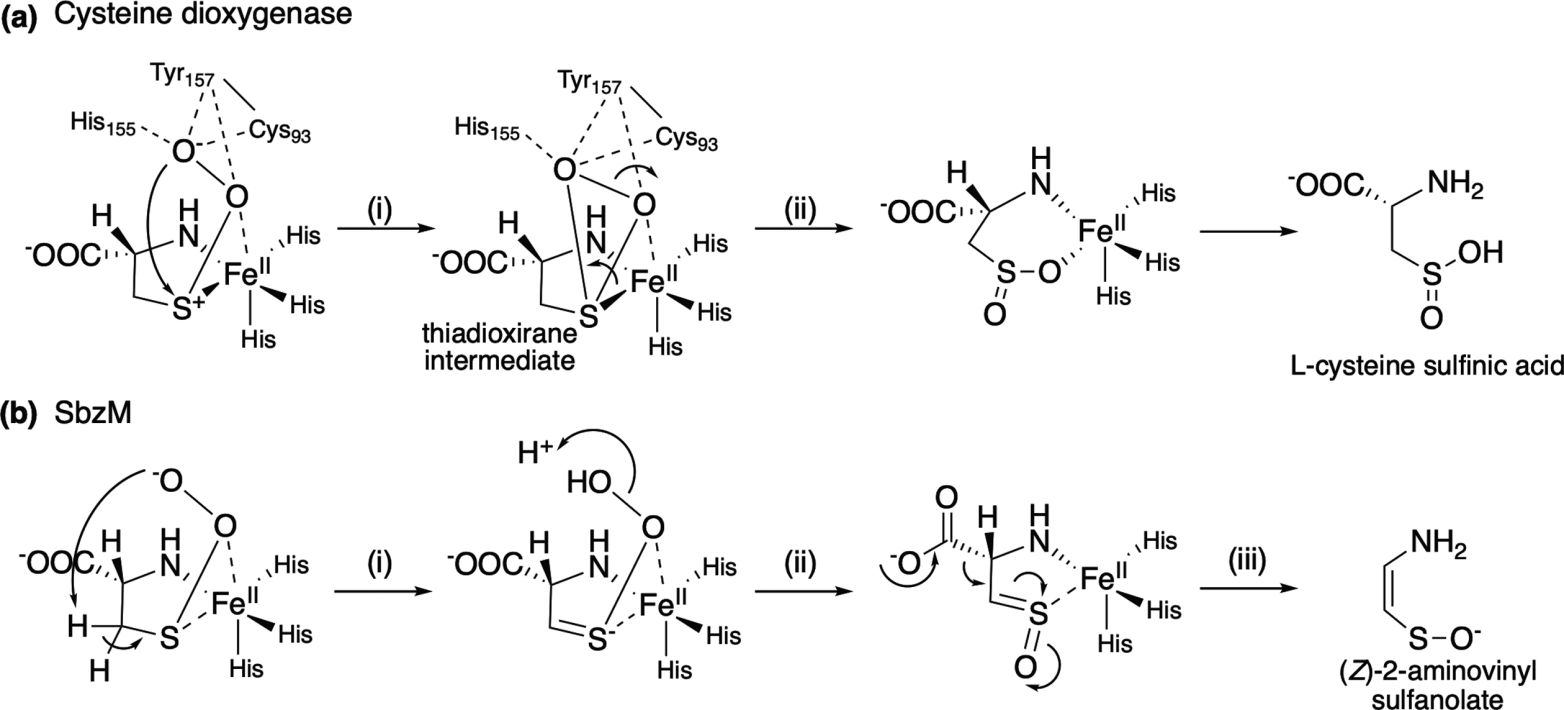
The hypothetical reaction mechanism of cysteine dioxygenase and SbzM. (a) The reaction mechanism of cysteine dioxygenase to produce l-cysteine sulfinic acid. (b) The hypothetical reaction mechanism of SbzM to produce (*Z*)-2-aminovinyl sulfanolate.

The known cupin-type enzyme cysteine dioxygenase also accepts l-cysteine, but produces l-cysteine sulfinic acid (Stipanuk et al., 2011). Mechanistically, it is proposed that the superoxide anion attacks the cationic sulfur to form the thiadioxirane intermediate (reaction i), which rearranges to l-cysteine sulfinic acid (reaction ii) (Fig. 6a) (Li et al., 2019). In contrast, the SbzM reaction was suggested to involve proton abstraction at the β-position of l-cysteine by the superoxide species to produce the intermediate with a C–S double bond (reaction i) as reported in the peptidyl-cysteine decarboxylase EpiD (Fig. 6b) (Blaesse et al., 2000). The electrons move from the sulfur to the oxygen atom to produce an instable sulfine intermediate (McCaw et al., 2016) (reaction ii) that spontaneously rearranges to the more stable (*Z*)-2-aminovinyl sulfanolate concomitantly with decarboxylation (reaction iii) (Fig. 6b). To shed light on the mechanistic differences in catalysis of SbzM to the related cysteine dioxygenase, simulation studies with models built from X-ray crystallographic data, together with spectroscopic experiments are required.

**Fig. 7.**
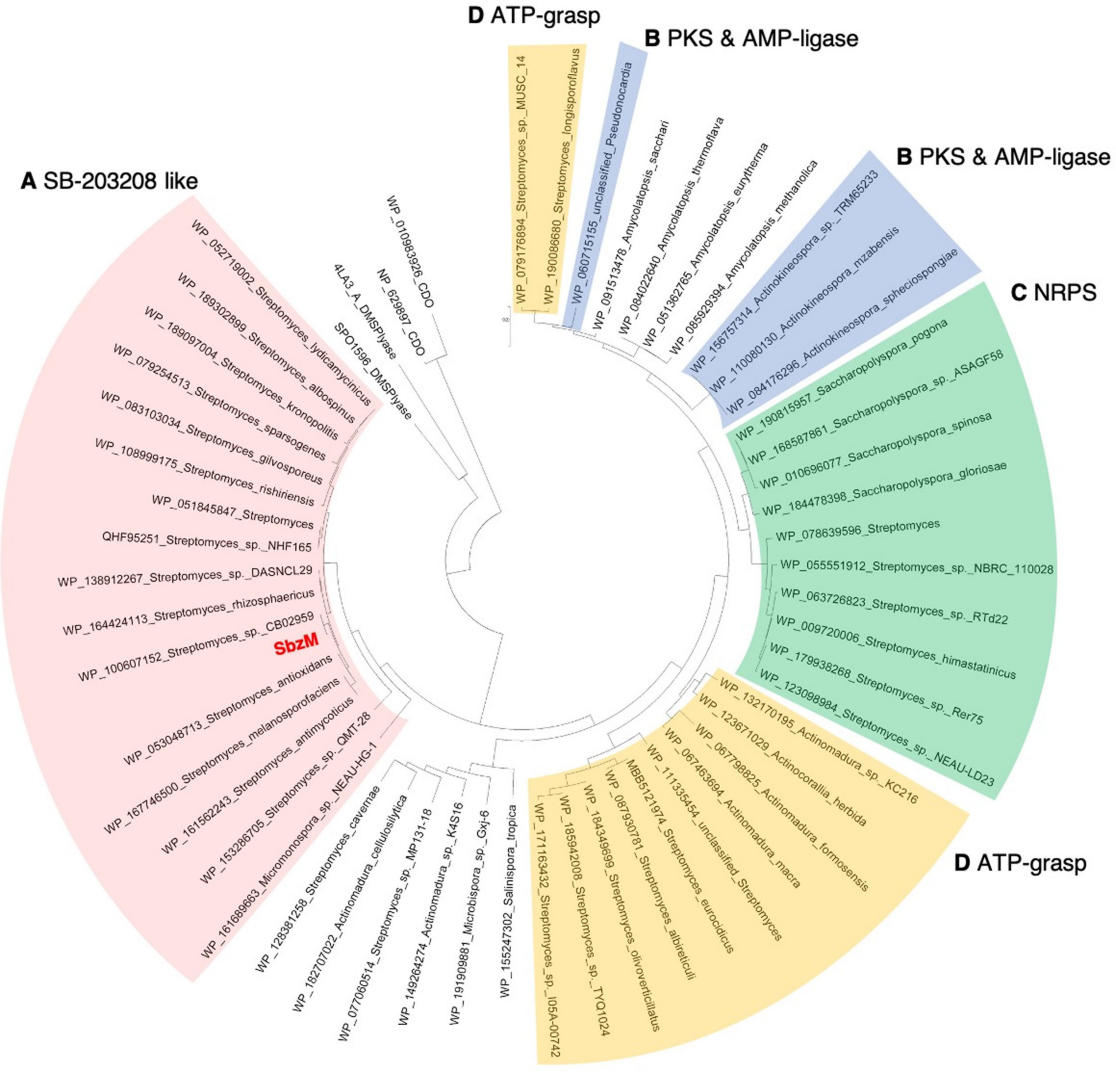
The phylogenetic tree analysis of SbzM homologs. Each biosynthetic gene is clustered with genes encoding SB-203208-type biosynthetic enzymes (A), PKS & AMP ligase (B), NRPS (C), and ATP-grasp enzyme (D).

SbzM homologs with high similarity (49.5–96.5% similarity) are distributed among actinomycetes such as *Streptomyces, Micromonospora, Amycolatopsis, Saccharopolyspora, Actinomadura, Actinokineospora, Actinocorallia, Microbiospora*, and *Pseudonocardia* (Fig. 7). Some of these homologs are distributed in *sbz*-like cluster (Fig. 7A). More interestingly, the other homologs are clustered with genes encoding polyketide synthase (PKS) and AMP-ligase possibly to load a substrate for PKS (Fig. 7B), non-ribosomal peptide synthetase (NRPS) genes (Fig. 7C), and ATP-grasp enzymes that form amide bond (Fig. 7D) (Ogasawara & Dairi, 2017; Walsh & Tang, 2017). So far, there is no knowledge about natural products derived from these clusters, making them promising targets for the discovery of novel sulfonamide-containing secondary metabolites.

## Conclusion

In this review, we summarized the knowledge on the biosynthesis of antibiotic sulfamate and sulfonamide natural products from actinomycetes. Although there are still several questions unanswered, our current understanding reveals the occurrence of distinct chemical strategies for S–N bond formation in known pathways. The sulfamate formation in ascamycin biosynthesis is proposed to be based on initial sulfate activation by anhydride formation (PAPS) and transfer of the sulfuryl group to the acceptor molecule by a sulfotransferase, followed by S–N bond formation by an unknown mechanism. The sulfonamide formation in xiamycin biosynthesis on the other hand is based on oxidation-induced radical formation by a flavin-dependent enzyme and the use of SO_2_ from the bacterial sulfur metabolism. The sulfonamide assembly in altemicidin, SB-203208, and SB-203207 biosynthesis utilizes cysteine and tailoring by a cupin-type oxygenase catalyzing an intriguing multistep reaction that involves oxidative decarboxylation, intramolecular nucleophilic substitution, and imine hydrolysis. The variety of these reactions are impressive examples for the versatile chemistries that have evolved in biosynthetic pathways toward highly potent secondary metabolites in actinomycetes. The strong and distinct bioactivities of the sulfamate and sulfonamide compounds make them privileged structures from Nature and novel strategies to diversify the family of these compounds is highly desirable. A detailed understanding of the underlying biosynthetic machineries will open the door to exploit them for the development of novel drugs or drug leads, biotechnological production of target compounds, or the development of new biocatalysts by protein engineering efforts.
